# Difficult intubation and outcome after out-of-hospital cardiac arrest: a registry-based analysis

**DOI:** 10.1186/s13049-015-0124-0

**Published:** 2015-06-06

**Authors:** Jan Wnent, Rüdiger Franz, Stephan Seewald, Rolf Lefering, Matthias Fischer, Andreas Bohn, Jörg W. Walther, Jens Scholz, Roman-Patrik Lukas, Jan-Thorsten Gräsner, the German Resuscitation Registry Study Group

**Affiliations:** Department of Anesthesiology and Intensive-Care Medicine, University Medical Center Schleswig-Holstein, Campus Luebeck, Ratzeburger Allee 160, Haus 13, 23538 Luebeck, Germany; Department of Anesthesiology and Intensive Care Medicine, European Medical School Oldenburg-Groningen, Oldenburg, Germany; Department of Anesthesiology and Intensive-Care Medicine, University Medical Center Schleswig-Holstein, Campus Kiel, Schwanenweg 21, 24105 Kiel, Germany; University of Witten/Herdecke, Faculty of Medicine, Institute for Research in Operative Medicine, Ostmerheimer Strasse 200, Haus 38, 51109 Cologne, Germany; Department of Anesthesiology and Intensive-Care Medicine, Klinik am Eichert, ALB.Fils-Kliniken, Eichertstrasse 3, 73035 Göppingen, Germany; City of Münster, Fire Department, York-Ring 25, 48159 Münster, Germany; Department of Anesthesiology and Intensive-Care Medicine, Münster University Hospital, Albert-Schweitzer-Campus 1, Gebäude A1, 48149 Münster, Germany; Institute for Prevention and Occupational Medicine, Ruhr-Universität Bochum, Buerkle de la Camp-Platz 1, 44789 Bochum, Germany

**Keywords:** Intubation, Out-of-hospital cardiac arrest, Resuscitation, Airwaymanagement

## Abstract

**Background:**

Airway management during resuscitation attempts is pivotal for treating hypoxia, and endotracheal intubation is the standard procedure. This German Resuscitation Registry analysis investigates the influence of airway management on primary outcomes after out-of-hospital cardiac arrest, in a physician-based emergency system.

**Methods:**

A total of 8512 patients recorded in the German Resuscitation Registry (2007–2011) were analyzed. The Return of Spontaneous Circulation After Cardiac Arrest (RACA) score was used to compare observed return of spontaneous circulation (ROSC) rates with the ROSC predicted by the score and to analyze factors influencing the primary outcome. Patients were classified into three groups: difficult intubation, impossible intubation, and a control group with normal airways.

**Results:**

The observed ROSC matched the predicted ROSC in the group with difficult airways. The impossible intubation group had lower ROSC rates (31.3 % vs. 40.5 %; *P* < 0.05). Impossible intubation was more frequent in men (OR 2.28; 95 % CI, 1.43–3.63; *P* = 0.001), young patients (OR 2.18; 95 % CI, 1.26–3.76; *P* = 0.005) and those with trauma (OR 2.22; 95 % CI, 1.01–4.85; *P* = 0.046). Fewer impossible intubations were reported when the emergency physicians were anesthesiologists (OR 0.65; 95 % CI, 0.44–0.96; *P* = 0.028). If a supraglottic airway device was not used in the impossible intubation group, the observed ROSC (18.0 %; 95 % CI, 7.4–28.6 %) was poorer than predicted (38.2 %) (*P* < 0.05).

**Conclusions:**

Outcomes after resuscitation attempts are poorer when endotracheal intubation is not possible. Predictive factors for impossible intubation are male gender, younger age, and trauma. Supraglottic airway devices should be used at an early stage whenever these negative factors are present.

**Electronic supplementary material:**

The online version of this article (doi:10.1186/s13049-015-0124-0) contains supplementary material, which is available to authorized users.

## Introduction and background

The quality of cardiopulmonary resuscitation (CPR) performed by emergency medical service (EMS) staff has been identified as an independent predictive factor for the outcome after out-of-hospital cardiac arrest [1]. The quality is related to the extent to which guidelines are implemented and how effectively they are translated into everyday routine work [2, 3]. CPR technique and the associated technical skills affect the primary outcome [4].

During resuscitation attampts, endotracheal intubation is still regarded as the “gold standard” for airway management. Endotracheal intubation should be performed during ongoing chest compression and should only be carried out by professionals who are well trained and experienced in this technique [5]. The aim of the present study was to investigate the influence of difficulties in endotracheal intubation on the primary outcome in patients with out-of-hospital cardiac arrest (OHCA) [6–9].

The primary outcome, defined as return of spontaneous circulation (ROSC) after OHCA, depends on several factors that have been previously studied [10–12]. Some of these influencing factors are fixed, due to the patient’s condition and the surrounding circumstances, and cannot be changed or influenced by the EMS [13]. These include age, gender, location of cardiac arrest, no-flow time, basic life support provided before the arrival of the EMS, presumable etiology, and the initial electrocardiography (ECG) rhythm [12, 14–17]. To allow comparison of the effects of different treatment strategies on the primary outcome, the ROSC after Cardiac Arrest (RACA) score was developed and published in 2011 [13].

In this retrospective registry-based study, the incidence of difficult and impossible intubation during resuscitation attempts in Germany and the impact on ROSC after OHCA were calculated. The impact of the expertise and specialization of the physician on the scene in relation to the incidence of difficult and impossible intubations and the use of supraglottic airway devices during difficult and impossible intubations and its impact on the primary outcome after OHCA were analyzed as well.

## Methods

The German Resuscitation Registry (GRR) is a national prospective database for both out-of-hospital and in-hospital cardiac arrest patients [18]. This study included 8512 patients with a “prehospital care data set”, in which prehospital technical information, timestamps, presumed etiology, resuscitation therapy, and the patient’s primary outcome were recorded from 2007 until 2011 in accordance with the Utstein style recommendations [19, 20].

Twenty-two emergency medical systems staffed by emergency physicians (the GRR Study Group) contributed to the study. The physicians on the scene were anesthetists, surgeons, and internists who had completed a special training program in emergency medicine.

The study design and publication were approved by the scientific committee of the GRR in the German Society of Anesthesiology and Intensive-Care Medicine, in compliance with current publication guidelines. The need to obtain informed consent from the patients was waived by the ethics committee of the University of Cologne, Faculty of Medicine (Record No. 11–014) and the ethics committee of the University of Kiel, Faculty of Medicine (Record No. D 432/13).

### Inclusion criteria

The present analysis includes patients with OHCA in whom cardiopulmonary resuscitation (CPR) was started. Out-of-hospital cardiac arrest was defined in accordance with the Utstein style criteria as a cessation of cardiac mechanical activity, confirmed by the absence of signs of circulation. Only data sets that provided the full information necessary for calculating the RACA score (age, location of cardiac arrest, first monitored ECG rhythm, witnessing, bystander CPR, suggested etiology, arrival time of EMS) were included in the analysis [13].

### Exclusion criteria

Patients with definite signs of death, patients with a do-not-attempt-resuscitation order, and patients presenting with injuries that were obviously associated with no chance of survival were excluded. Incomplete data sets were also excluded from further analysis. Children and adolescents up to the age of 18 were also excluded from the present analysis due to their special physiologic and pathophysiologic conditions and the small sample size (*n* = 191).

### Definitions of difficulties in endotracheal intubation

Difficult endotracheal intubation was defined as any problems occurring during the insertion of an endotracheal tube, even if it was finally successful. Due to the definitions established in the Utstein style and GRR data set, no distinction was made between numbers of attempts or reasons for these difficulties.

Impossible endotracheal intubation was defined as an unsuccessful attempt to insert an endotracheal tube irrespective of the number of attempts.

### Data management

The GRR is a prospective web-based database used to record all EMS- and emergency physician–related resuscitation efforts. The GRR’s data management system has been shown to be consistent with the Utstein style, and its control mechanisms guarantee data collection and data quality in connection with out-of-hospital (18) and in-hospital cases of cardiac arrest [21].

### End points

In accordance with the Utstein definition, the primary outcome was the return of spontaneous circulation (ROSC), defined as a palpable pulse for more than 20 s [22].

### RACA score

The RACA score is a simple tool for calculating a predicted outcome rate (the ROSC rate) on the basis of independent variables that cannot be changed by the EMS. The exact calculation of the score is described elsewhere [13].

In addition to the Utstein style and RACA criteria, the impact of the criteria “qualification” and “specialty” of the physician on the scene and the use of supraglottic airway devices (SGD) was analyzed. It was not possible to provide information about the specific type of alternative airway used, as the EMS may use different airway devices that are summarized under “supraglottic airway” when recorded in the “prehospital care” data set.

### Statistical analysis

On the basis of the prospectively recorded multicenter data sets, a retrospective analysis was carried out in relation to effects of the criteria “difficult intubation” and “impossible intubation” on the primary resuscitation outcome, as measured by the ROSC rate. The relationship between the criteria “difficult intubation” and “impossible intubation” and the RACA score was also analyzed.

The data were divided into three subgroups: group 1, all data sets with “difficult intubation”; group 2, all data sets with “impossible intubation”; and group 3, a control group containing all data sets in which no problems with intubation were recorded.

Statistical analysis of the binary and categorical variables was carried out using the chi-square test and Fisher’s exact test, as appropriate. For the variables of age and time, the *U* test and Kruskal–Wallis tests were used. *P* < 0.05 was considered to indicate statistical significance.

In a second step, the impact of the Utstein style criteria on the incidence of impossible intubation was analyzed by univariate analysis. All variables yielding *P* < 0.10 in the univariate analysis were subsequently included in a binary logistic regression analysis (Table 1).

**Table 1:** Predictors of impossible intubation. (forwards stepwise binary logistic regression analysis) Variables not shown in equation: Resident physician in internal medicine

	Regression-coefficient	SE	*P*-value	OR (95 % CI)
Gender male	0.82	0.24	*p* = 0.001	2.28 (1.43–3.63)
Age < 80 years	0.78	0.28	*p* = 0.005	2.18 (1.26–3.76)
presumed etiology			*p* = 0.034	
presumed etiology – trauma	0.80	0.40	*p* = 0.046	2.22 (1.01–4.85)
presumed etiology – hypoxia	0.49	0.27	*p* = 0.067	1.63 (0.97–2.74)
board certified physician in Anesthesia	−0.43	0.20	*p* = 0.028	0.65 (0.44–0.96)
constant	−5.22	0.32	*p* < 0.001	

All statistical analyses were performed with SPSS, version 18 (SPSS Inc., Chicago, Illinois, USA).

## Results

**Fig. 1:**
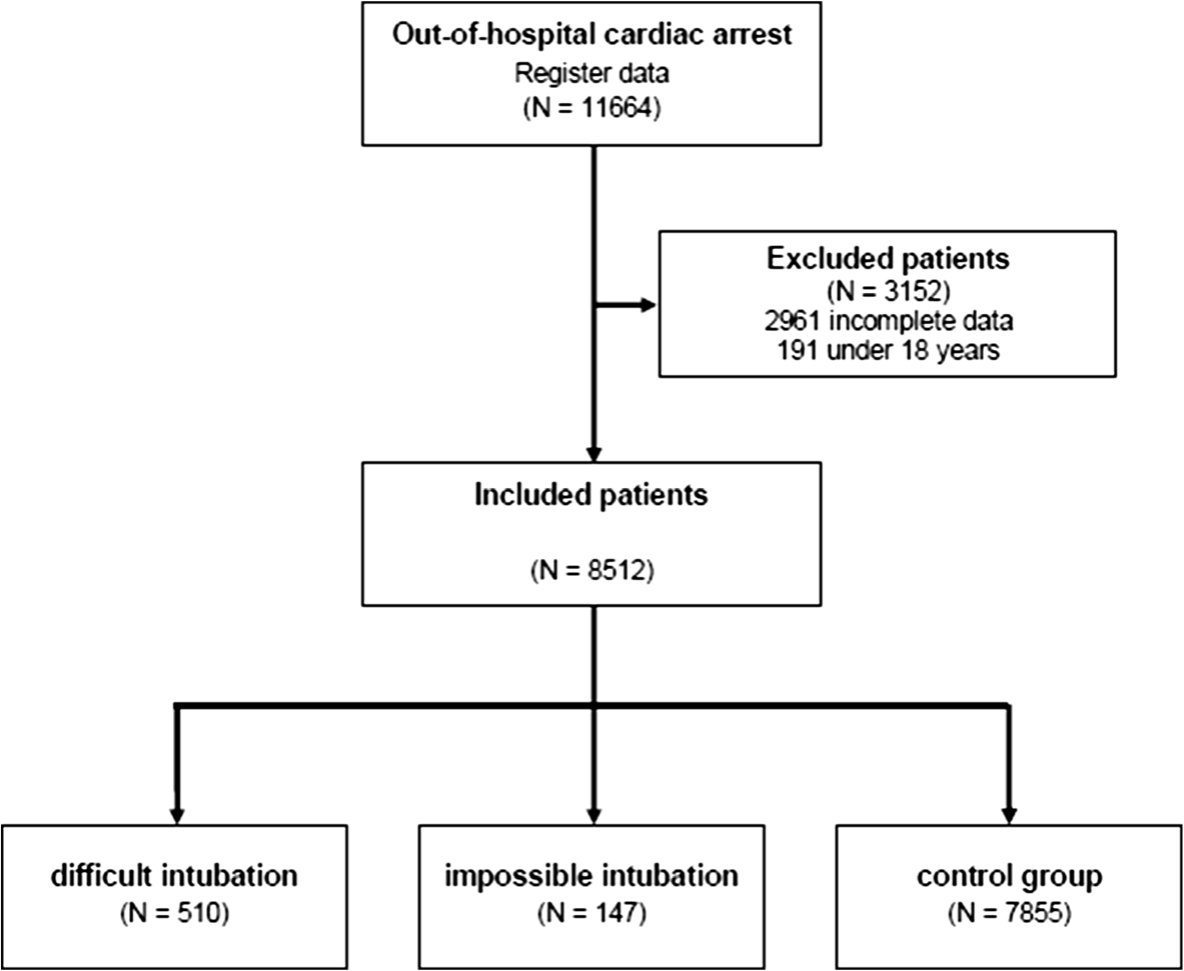
Flow chart for study patients with out-of-hospital cardiac arrest between January 2007 and October 2011. CPR,cardiopulmonary resuscitation

Between January 2007 and October 2011, a total of 11,664 patients were documented in the GRR following out-of-hospital cardiac arrest. A total of 3152 data sets were excluded from the analysis due to missing information (*n* = 2961), age below 18 years (*n* = 191). Of the remaining 8512 patients with OHCA, 41.9 % achieved ROSC on the scene (*n* = 3565) and 45.4 % were admitted to hospital (*n* = 3864) with ongoing CPR (*n* = 613) or ROSC (*n* = 3251). Figure 1 shows a flow diagram for the study patients.

A total of 510 patients with difficult endotracheal intubation were documented and intubation was impossible in 147 patients. No difficulties were recorded in relation to airway management in the remaining 7855 patients (normal endotracheal intubation). The characteristics of the different groups are shown in Table 2.

**Table 2:** Comparison of Study Patients with Out-of-Hospital Cardiac Arrest Documented in the German Resuscitation Registry Relative to Intubation Problems (January 2007–October 2011)

	Difficult endotracheal intubation	Impossible endotracheal intubation	Normal endotracheal intubation (control group)	*P*-values	Test method
n	510	147	7855		
Gender male	378 (74.1 %)	117 (79.6 %)	5194 (66.1 %)	*p* < 0.001	Chi^2^
Age in years (MD +/− SD)	64.5 +/−14.9	64.1 +/−14.9	68.7 +/−15.2	*p* < 0.001	Kruskal-Wallis
Age > 80 years	85 (16.7 %)	18 (12.2 %)	1973 (25.1 %)	*p* < 0.001	Chi^2^
Location					
- At home	325 (63.7 %)	97 (66.0 %)	5271 (67.1 %)	0.028	Chi^2^
- Nursing home	30 (5.9 %)	7 (4.8 %)	611 (7.8 %)		
- Doctor’s office	5 (1.0 %)	4 (2.7 %)	147 (1.9 %)		
- Public place	111 (21.8 %)	32 (21.8 %)	1357 (17.3 %)		
- Medical institution	11 (2.2 %)	2 (1.4 %)	191 (2.4 %)		
- Others	28 (5.5 %)	5 (3.4 %)	278 (3.5 %)		
Presenting rhythm					
- Ventricular Fibrillation	154 (30.2 %)	40 (27.2 %)	2235 (28.5 %)	0.274	Chi^2^
- EMD	79 (15.5 %)	17 (11.6 %)	1345 (17.1 %)		
- Asystole	277 (54.3 %)	90 (61.2 %)	4275 (54.4 %)		
Witnessed					
- None	153 (30.0 %)	60 (40.8 %)	3046 (38.8 %)	*p* < 0.001	Chi^2^
- Lay people	309 (60.6 %)	70 (47.6 %)	3952 (50.3 %)		
- Professionals	48 (9.4 %)	17 (11.6 %)	857 (10.9 %)		
Bystander CPR	125 (24.5 %)	29 (19.7 %)	1173 (14.9 %)	*p* < 0.001	Chi^2^
Presumed etiology					
- Cardial	375 (73.5 %)	109 (74.1 %)	6360 (81.0 %)	*p* < 0.001	Chi^2^
- Trauma	21 (4.1 %)	7 (4.8 %)	181 (2.3 %)		
- Hypoxia	66 (12.9 %)	20 (13.6 %)	698 (8.9 %)		
- Intoxikation	6 (1.2 %)	5 (3.4 %)	114 (1.5 %)		
- Other not cardial	42 (8.2 %)	6 (4.1 %)	502 (6.4 %)		
use of SGD	152 (29.8 %)	97 (66.0 %)	530 (6.7 %)	*p* < 0.001	Chi^2^
Arrest to EMS arrival time (MD +/−SD)	9.1 +/− 5.8	8.3 +/−5.6	8.7 +/−6.0	0.092	Kruskal-Wallis
observed ROSC	222 (43.5 %)	46 (31.3 %)	3297 (42.0 %)	0.025	Chi^2^
(95 % CI)	(39.2–47.8 %)	(23.8–38.8 %)	(40.9–43.1 %)		
expected ROSC (RACA)	42.0 %	40.5 %	39.8 %		

CPR, cardiopulmonary resuscitation; EMD, electromechanical dissociation; EMS, emergency medical system; RACA, ROSC after cardiac arrest score; ROSC, return of spontaneous circulation; SGD, supraglottic airway device

The incidence of difficult or impossible intubation remained relatively constant. The use of supraglottic airway devices (SGD) has increased continuously since 2007.

### Patients with difficult intubation

**Fig. 2:**
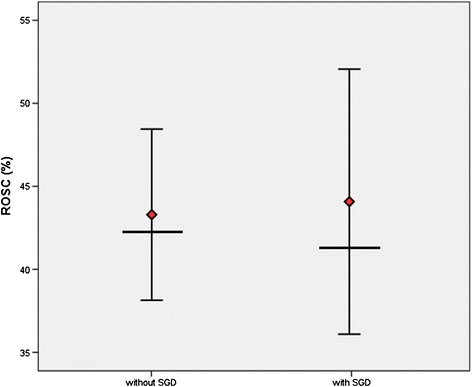
Mean rate of observed return of spontaneous circulation (ROSC) (95 % confidence intervals) in comparison with the predicted rate of return of spontaneous circulation (*black bar*) in difficult intubation group. ROSC, return of spontaneous circulation; SGD, supraglottic airway device

ROSC was achieved in 43.5 % of cases (*n* = 222; 95 % CI, 39.2 to 47.8 %). The expected ROSC (= RACA score) in this group was 42.0 %. SGDs were used in 29.8 % of the cases (*n* = 152 of 510). If SGDs were used, ROSC was achieved in 44.1 % (*n* = 67; 95 % CI, 36.2 to 52.0 %), in comparison with a predicted ROSC of 41.3 % according to the RACA score (n.s.). If SGD were not used, ROSC was achieved in 43.3 % of cases (*n* = 155; 95 % CI, 38.2 to 48.4 %). The predicted ROSC in this group was 42.3 % (n.s.) (Fig. 2). Qualification of the physicians on the scene is shown in Table 3.

**Table 3:** Qualification of the physician on scene relative to intubation problems

	Difficult endotracheal intubation	Impossible endotracheal intubation	Normal endotracheal intubation (control group)
**n**	510	147	7855
**qualification**			
- resident physicians	224 (45.3 %)	61 (45.9 %)	3015 (41.2 %)
- Board-certified physicians	270 (54.7 %)	72 (54.1 %)	4308 (58.8 %)
- unkown	16	14	532
**field**			
- anesthetists	271 (55.0 %)	74 (55.2 %)	4829 (66.1 %)
- internists	129 (26.2 %)	36 (26.9 %)	1428 (19.5 %)
- surgeons	61 (12.4 %)	17 (12.7 %)	701 (9.6 %)
- other specialties	32 (6.5 %)	7 (5.2 %)	350 (4.8 %)
- unkown	17	13	547

### Patients with impossible intubation

ROSC was achieved in 31.3 % of cases (*n* = 46/147; 95 % CI, 23.8 to 38.8 %) in comparison with a predicted ROSC rate of 40.5 % (*P* < 0.05). An alternative airway was used in 66.0 % of these cases (*n* = 97/147).

**Fig. 3:**
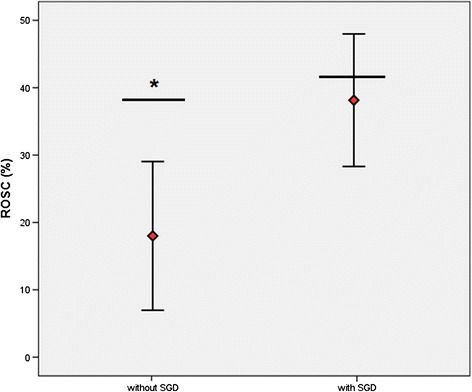
Mean rate of observed return of spontaneous circulation (ROSC) (95 % confidence intervals) in comparison with the predicted rate of return of spontaneous circulation (*black bar*) in impossible intubation group. ROSC, return of spontaneous circulation; SGD, supraglottic airway device

If a SGD was used, ROSC was observed in 38.1 % (*n* = 37; 95 % CI, 28.5 to 47.8 %). In accordance with the RACA score, ROSC would have been expected in 41.6 % (n.s.). If an SGD was not used, ROSC was observed in 18.0 % (*n* = 9; 95 % CI, 7.4 to 28.6 %). In accordance with the RACA score, ROSC would have been expected in 38.2 % of these cases (*P* < 0.05) (Fig. 3).

Significant predictive factors for impossible intubation identified in the binary logistic regression analysis are listed in Table 1.

### Control group with normal airway

ROSC was observed in 42.0 % of cases (*n* = 3297/7855; 95 % CI, 40.9 to 43.1 %). According to the RACA score, ROSC would have been expected in 39.8 % of these cases (Additional file 1).

## Discussion

In a total of 8512 resuscitation attempts analyzed, intubation was difficult in 6.0 % of all the patients in the registry and impossible in 1.7 %. These figures were analyzed in the context of other publications on the influence of difficult or impossible intubation on the patient’s [23, 24]. In prehospital emergency medicine, the incidence of a difficult airway may vary widely and may depend on the type of emergency service being analyzed. The incidence of difficult or impossible intubation is higher in reports from countries with paramedic-based emergency services. Cobas and coworkers found that 31 % of 203 patients with prehospital intubation met the criteria for unsuccessful intubation [24]. Wang et al. reported a success rate of only 77 % in 10,356 attempted prehospital intubations [25]. In their study, 4482 patients were intubated during ongoing resuscitation, with an intubation success rate of 78 %.

Breckwoldt and coworkers reported an incidence of difficult intubation of 13 % in 276 intubations analyzed, a rate similar to that observed in the present study in the physician-based EMS in Germany [26]. Intubation failed in 1.4 % of patients. Intubations were most often attempted during cardiac arrest (63.8 % of all patients) [23]. This was also observed in other systems in Germany. In another physician-based EMS, Chenaitia et al. noted a 6 % incidence of more than two intubation attempts or a need for an alternative airway device in 239 prehospital intubations [27]. Adnet et al. reported a 99.1 % success rate in 691 prehospital intubations [28]. Although a difficult airway was present in 10.8 % of the cases, the intubation failed in only 0.9 % of the patients. Patients with cardiac arrest formed the largest proportion of the patient population enrolled (48.2 %).

The incidence of difficult or even impossible endotracheal intubation is markedly higher during EMS operations in comparison with clinical anesthesia. In clinical anesthesia, Burkle and coworkers noted intubation difficulties in 0.5 % of anesthetic procedures (186/37,482), while 0.4 % of the attempted intubations failed and airway management was achieved with a supraglottic airway device [29].

To make things worse, EMS staff on the scene often do not have the same level of training and skills as hospital staff, who are routinely acquainted with the tasks required or can call for additional help and resources very quickly. EMS physicians from subspecialties other than anesthesia may therefore need further training or provision of alternative airway devices.

The observed ROSC rate in the impossible intubation group was significant reduced compared to the predicted ROSC rate according to the RACA score in these groups. It was found that difficulties in endotracheal intubation have no influence on the ROSC when the endotracheal tube is in the end successfully placed. However, impossible endotracheal intubation was followed by a significant reduction in the ROSC rate.

Difficult or impossible intubation was more frequent in male patients. This proportion was significantly lower in the control group. Male gender was an independent predictive factor for impossible intubation. An association between male gender and difficult intubation was also reported by Timmermann and coworkers, with 79 % of the male patients having a difficult airway, while males accounted for only 64 % in the overall population [9]. In a study on prehospital airway management, Thierbach and coworkers reported that 18.3 % of males but only 9.5 % of females needed several intubation attempts [30].

Age had a significant impact on the incidence of a difficult or impossible intubation in the present study. Age below 80 is an independent predictive factor for an impossible intubation. This is in contrast to the findings reported by Chenaitia et al., in which the mean age in the group with a difficult airway was 64 years, in comparison with a mean of 54 in patients with an uncomplicated airway [27].

In the present analysis, CPR due to trauma was an independent predictive factor for impossible intubation. Few data have been published on the incidence of difficult or impossible intubation during CPR, trauma, and hypoxia. Timmermann et al. reported a higher incidence of difficult airways in patients after trauma, but did not explicitly mention the combination of CPR and trauma [9]. Thierbach and coworkers found that intubation was successful at the first attempt in 70 % of patients after trauma, in comparison with 85 % of patients without trauma [30].

In this analysis, the level of training of the physician on the scene was found to have a significant impact on the incidence of impossible intubation. The multivariate analysis showed that physicians with board certification in anesthesia have a positive impact in preventing situations of impossible intubation and are able to counteract all the other negative factors identified in the study.

Several authors have reported on the impact of physicians’ level of training on the outcome in prehospital emergency medicine. Breckwoldt and coworkers also reported that difficult intubation was significantly less frequent with board-certified anesthetists in comparison with board-certified internists with experience in intensive care [26]. In this context, Timmermann reported that 18 % of emergency physicians who were not anesthetists had performed less than 20 intubations in controlled conditions in hospital [7]. This finding is particularly important, as nearly 42 % of nonanesthetist emergency physicians have no experience with any type of supraglottic airway device and 55.5 % have no experience in obtaining surgical access to the airway.

The use of a supraglottic airway device in the difficult intubation group had no impact on the primary outcome in the present analysis. By contrast, if a supraglottic airway device was *not* used after impossible intubation, the observed ROSC was significantly poorer than that predicted by the RACA score. The use of an SGD reduces the negative impact on the ROSC if impossible intubation occurs. According to the RACA/ROSC analyses, use of an SGD in this situation was able to overcome the negative impact of impossible intubation.

As the study is registry-based, it was not possible to obtain information about the specific devices and manufacturers of SGDs; several different supraglottic devices were used.

Schalk and coworkers reported on the safety of the laryngeal tube in a German EMS in which the device was used both by physicians and nonphysicians. Ventilation was successful in all 157 patients, and the device was used both after failed intubation and as a primary airway [31, 32].

A continuing increase in the use of supraglottic airway devices has been observed since 2007, and they are now being used during CPR significantly more often in comparison with 2008. Data are not available on the proportion of cases in which the use of a supraglottic airway for ventilation failed, leading to the documentation of an impossible intubation, and it can therefore not be concluded that using a supraglottic airway device is able to prevent failed intubation. In 2011, more than 20 % of resuscitated patients did not undergo endotracheal intubation as the primary airway. This suggests that the use of supraglottic airway devices is not restricted to difficult airway management and that they are increasingly being used as a primary airway. Further research is needed in order to assess the positive or negative impact of routine use of SGDs. In some studies, SGD use has been reported to have a negative impact on the outcome for cardiac arrest victims [33].

## Limitations

The GRR is based on voluntary participation by emergency services and hospitals and covers a population of 12 million. It is not capable of providing complete data on OHCA incidents and resuscitation attempts throughout Germany. Although there is therefore some degree of uncertainty with regard to the representativeness of the registry, it still reflects current practice throughout the country in both rural areas and large cities, with different emergency medical system patterns. Voluntary registration and documentation by 22 medical emergency systems providing data for the prehospital care data sets is likely to be associated with a risk of inclusion bias in the present study. This is a problem typically associated with all registry-based studies [34].

The limited number of 8512 patients included resulted from a strict limitation to patients with complete prehospital care data sets. ROSC was selected as the primary end point due to the known impact of postresuscitation care on the secondary outcome of CPR, as measured by hospital discharge findings.

The classification of cases as representing difficult or impossible endotracheal intubation was made by the physician on the scene and could not be verified by someone else in the EMS setting.

## Conclusions

The outcome after resuscitation attempt is poorer in patients in whom endotracheal intubation is not possible. Predictive factors for impossible intubation identified in the present study are male gender, younger age, and trauma. Supraglottic airway devices should be used at an early stage whenever these negative factors are present and a board-certified anesthetist is not on the scene.

## Additional file

Additional file 1: Table S1. Comparison of included and excluded cases. (CPR, cardiopulmonary resuscitation; EMD, electromechanical dissociation; EMS, emergency medical system; ROSC, return of spontaneous circulation).

## Abbreviations

CI: Confidence interval(s)
ECG: Electrocardiogram
EMS: Emergency medical service
GRR: German Resuscitation Registry
OHCA: Out-of-hospital cardiac arrest
OR: Odds ratio
RACA: ROSC after cardiac arrest (score)
ROSC: Return of spontaneous circulation
SGD: Supraglottic airway device
