# Supplementary material for: Difficult intubation and outcome after out-of-hospital cardiac arrest: a registry-based analysis
Source: Scand J Trauma Resusc Emerg Med. 2015 Jun 6;23:43. doi: 10.1186/s13049-015-0124-0 (PMC4457979; doi:10.1186/s13049-015-0124-0)
Supplement: Additional file 1: Table S1. — Comparison of included and excluded cases. (CPR, cardiopulmonary resuscitation; EMD, electromechanical dissociation; EMS, emergency medical system; ROSC, return of spontaneous circulation). [file 13049_2015_124_MOESM1_ESM.docx]

Table S1: Comparison of included and excluded cases. (CPR, cardiopulmonary resuscitation; EMD, electromechanical dissociation; EMS, emergency medical system; ROSC, return of spontaneous circulation)

|  | **included datasets** | **excluded datasets** | **P-values** | **OR**  **(95% CI)** | **Test method** |
| --- | --- | --- | --- | --- | --- |
| **n** | 8512 | 3152 |  |  |  |
| **Gender male** | 5689 (66.8%) | 1896 (62.5%) | p<0.001 | 1.21 (1.11-1.32) | Fisher exact |
| **Age in years (MD +/- SD)** | 68.4 +/- 15.2 | 65.2 +/- 21.0 | 0.003 |  | U-Test |
| **Age > 80 years** | 2076 (24.4%) | 753 (24.4%) | 0.999 | 1.00 (0.91-1.10) | Fisher exact |
| **Location** |  |  |  |  |  |
| - At home | 5693 (66.9%) | 1985 (65.4%) | 0.003 |  | Chi² |
| - Nursing home | 648 (7.6%) | 213 (7.0%) |  |  |  |
| - Doctor’s office | 156 (1.8%) | 90 (3.0%) |  |  |  |
| - Public place | 1500 (17.6%) | 570 (18.8%) |  |  |  |
| - Medical institution | 204 (2.4%) | 78 (2.6%) |  |  |  |
| - Others | 311 (3.7%) | 101 (3.3%) |  |  |  |
| **Presenting rhythm** |  |  |  |  |  |
| - Ventricular Fibrillation | 2429 (28.5%) | 498 (24.6%) | p<0.001 |  | Chi² |
| - EMD | 1441 (16.9%) | 309 (15.3%) |  |  |  |
| - Asystole | 4642 (54.5%) | 1214 (60.1%) |  |  |  |
| **Witnessed** |  |  |  |  |  |
| - None | 3259 (38.3%) | 1288 (40.9%) | p<0.001 |  | Chi² |
| - Lay people | 4331 (50.9%) | 1127 (35.8%) |  |  |  |
| - Professionals | 922 (10.8%) | 737 (23.4%) |  |  |  |
| **Bystander CPR** | 1327 (15.6%) | 440 (14.0%) | 0.030 | 0.88 (0.78-0.99) | Fisher exact |
| **Presumed etiology** |  |  |  |  |  |
| - Cardial | 6844 (80.4%) | 2302 (73.0%) | p<0.001 |  | Chi² |
| - Trauma | 209 (2.5%) | 150 (4.8%) |  |  |  |
| - Hypoxia | 784 (9.2%) | 337 (10.7%) |  |  |  |
| - Intoxikation | 125 (1.5%) | 47 (1.5%) |  |  |  |
| - Other not cardial | 550 (6.5%) | 316 (10.0%) |  |  |  |
| **Internal medicine** |  |  |  |  |  |
| - resident | 566 (7.2%) | 170 (6.2%) | 0.090 | 0.85 (0.72-1.02) | Fisher exact |
| - board certified | 1012 (12.8%) | 367 (13.4%) | 0.469 | 1.05 (0.92-1.19) | Fisher exact |
| **Surgery** |  |  |  |  |  |
| - resident | 346 (4.4%) | 106 (3.9%) | 0.250 | 0.88 (0.70-1.09) | Fisher exact |
| - board certified | 420 (5.3%) | 237 (8.6%) | p<0.001 | 1.68 (1.42-1.98) | Fisher exact |
| **Anesthesia** |  |  |  |  |  |
| - resident | 2306 (29.3%) | 689 (25.1%) | p<0.001 | 0.81 (0.74-0.90) | Fisher exact |
| - board certified | 2846 (36.1%) | 990 (36.1%) | 1.000 | 0.99 (0.91-1.09) | Fisher exact |
| **other medicine fields** |  |  |  |  |  |
| - resident | 57 (0.7%) | 32 (1.2%) | 0.035 | 1.62 (1.05-2.50) | Fisher exact |
| - board certified | 327 (4.1%) | 151 (5.5%) | 0.004 | 1.35 (1.10-1.64) | Fisher exact |
| **difficult intubation** | 659 (7.7%) | 218 (6.9%) | 0.097 |  | Chi² |
| **impossible intubation** | 147 (1.7%) | 42 (1.3%) |  |  |  |
| **Arrest to EMS arrival time (MD +/- SD)** | 8.7 +/- 6.0 | 6.5 +/- 6.3 | p<0.001 |  | U-Test |
| **observed ROSC** | 3565 (41.9%) | 1306 (44.4%) | 0.018 | 1.11 (1.02-1.21) | Fisher exact |
